# Non-linear associations between healthy Nordic foods and all-cause mortality in the NOWAC study: a prospective study

**DOI:** 10.1186/s12889-022-12572-8

**Published:** 2022-01-25

**Authors:** Torill M. Enget Jensen, Tonje Braaten, Bjarne K. Jacobsen, Guri Skeie

**Affiliations:** 1 Department of Community Medicine, Faculty of Health Sciences, UiT The Arctic University of Norway, Pb 6070 Langnes, NO-9037 Tromsø, Norway; 2 Department of Community Medicine, Faculty of Health Sciences, Centre for Sami Health Research, UiT The Arctic University of Norway, Tromsø, Norway

**Keywords:** Healthy Nordic diet, Sustainable diet, Fatty fish, Lean fish, Low-fat dairy, Wholegrains, Fruits and vegetables, All-cause mortality, Non-linear, Cohort study

## Abstract

**Background:**

The shape of the associations between intake of foods basic in a healthy Nordic diet and long-term health is not well known. Therefore, we have examined all-cause mortality in a large, prospective cohort of women in Norway in relation to intake of: Nordic fruits and vegetables, fatty fish, lean fish, wholegrain products, and low-fat dairy products.

**Methods:**

A total of 83 669 women who completed a food frequency questionnaire between 1996 and 2004 were followed up for mortality until the end of 2018. Cox proportional hazards regression models were used to examine the associations between consumption of the Nordic food groups and all-cause mortality. The Nordic food groups were examined as categorical exposures, and all but wholegrain products also as continuous exposures in restricted cubic spline models.

**Results:**

A total of 8 507 women died during the 20-year follow-up period. Nordic fruits and vegetables, fatty fish and low-fat dairy products were observed to be non-linearly associated with all-cause mortality, while higher intake of lean fish and wholegrain products reduced all-cause mortality. Intake levels and hazard ratios (HR) and 95% confidence intervals (CI) associated with lowest mortality were approximately 200 g/day of Nordic fruits and vegetables (HR 0.83 (95% CI: 0.77–0.91)), 10–20 g/day of fatty fish (10 g/day: HR 0.98 (95% CI: 0.94–1.02)) and 200 g/day of low-fat dairy products (HR 0.96 (95% CI: 0.81–1.01)) compared to no consumption. Consumption of fatty fish ≥ 60 g/day compared to no intake statistically significantly increased the mortality (60 g/day: HR 1.08 (95% CI: 1.01–1.16)), as did consumption of low-fat dairy products ≥ 800 g/day compared to no intake (800 g/day: HR 1.10 (95% CI: 1.02–1.20)). After stratification by smoking status, the observed association between Nordic fruits and vegetables and all-cause mortality was stronger in ever smokers.

**Conclusion:**

The associations between intake of foods basic in healthy Nordic diets and all-cause mortality may be non-linear. Therefore, assumptions of linear associations between traditional Nordic food groups and health outcomes could lead to wrong conclusions in analyses of healthy Nordic diets.

**Supplementary Information:**

The online version contains supplementary material available at 10.1186/s12889-022-12572-8.

## Background

Over the past decade there has been a movement towards health-promoting regional and environmentally friendly diets, and healthy Nordic diets have gained much attention in this context [1–8]. Healthy Nordic diets can be described as dietary patterns with emphasis on foods that have traditionally been used and cultivated in the Nordic region, such as fish, wholegrains like rye and oats, root vegetables, cabbages, fruits like apples and pears, rapeseed oil and, to a varying degree, including low-fat dairy products [1, 2].

In a previous study on healthy Nordic diet and mortality by Olsen et al., it was concluded that traditional Nordic foods should be considered in public health recommendations [1]. Optimal intake levels of traditional Nordic foods, and the ideal composition of healthy Nordic diets for long-term health are, however, uncertain. Subsequent studies have supported the results by Olsen et al. and linked high compliance with healthy Nordic diets to longevity in populations across Nordic countries, and to reduced risk of cardiovascular diseases, type 2 diabetes, and colorectal cancer [3–8]. The evidence is, however, not conclusive [9–12].

The heterogeneity of cut-off points used to classify intake level of foods included in healthy Nordic diet scores might be the reason for failure to identify credible evidence for health benefits of a healthy Nordic diet [12]. Differences in cut-off points between studies also create confusion for public health recommendations. Another dilemma with combined diet scores, such as those commonly used to measure adherence to healthy Nordic diets, is the assumption that they follow a linear scale, while dose–response relationships between foods and health-outcomes can be non-linear [13].

It is therefore relevant to examine potentially non-linear associations between food groups basic in healthy Nordic diets, and long-term health. Hence, the aim of this study is to evaluate the shape of the associations between the intake of Nordic fruits and vegetables, fatty fish, lean fish, wholegrain products, and low-fat dairy products and all-cause mortality, using a modelling tool that allows non-linear relationships.

## Materials and methods

### Study design and setting

The design of the Norwegian Women and Cancer Study (NOWAC) has been described in detail previously [14]. Briefly put, a random sample of 172 000 women drawn from the Norwegian National Population Registry was enrolled in two waves from 1991 to 2007. Participants completed a mailed, self-administered baseline questionnaire including questions about anthropometric, sociodemographic, dietary, reproductive, and lifestyle factors. Follow-up questionnaires were collected over approximately 6-year intervals after recruitment.

The sample for this prospective cohort study included 101 316 women aged 41–76 who completed a food frequency questionnaire (FFQ) during baseline mailing (waves 1996–1997 and 2003–2004; response rates of 57% and 48%, respectively), or during the first follow-up (wave 1991–1992 enrolment did not cover FFQ data; a response rate of 81%). Women with no follow-up (*n* = 16) were excluded. We further excluded women with implausible daily energy intake (< 2 500 kJ (*n* = 1 033) or > 15 000 kJ (*n* = 141)), and women with missing information on the following variables: body mass index (BMI) (*n* = 2 272), physical activity (*n* = 8 548), smoking habits (*n* = 1 407), and education (*n* = 4 230), leaving a total number of 83 669 women for the present analysis.

### Assessment of Nordic foods intake

Diet was assessed using validated, semi-quantitative food frequency questionnaires (FFQ) with approximately 85 frequency items [15–17]. A representative sample of the questionnaires used has previously been published [18]. The FFQ was designed to measure the typical diet during the past year with special emphasis on fish consumption. The response options were given with four to seven frequency categories ranging from never/seldom to six or more per week. Portion sizes for some food items were provided as natural (e.g., number of carrots) or household units (e.g., tablespoons).

The Norwegian Weight and Measurement Table with standardised portion sizes and weights was used to convert the consumption of food items to grams [19], and information about the nutrient content in foods was obtained from the Norwegian Food Composition Database [20]. The calculations of daily intake of food items, energy and nutrients were made using a statistical syntax in SAS (SAS Institute Inc., Cary, NC, USA) developed at the Department of Community Medicine, University of Tromsø, for the NOWAC cohort. Missing frequency values were treated as no consumption, and missing portion sizes were set to the smallest portion size alternative.

We have considered consumption of five traditional Nordic food groups as exposure of interest, selected to reflect components of a healthy Nordic diet [1, 2]; Nordic fruits and vegetables (apples/pears, broccoli/cauliflower, cabbage, carrots, swede); fatty fish classified as fish with ≥ 4% fat in the meat (salmon, trout, herring, mackerel); lean fish containing < 4% fat in the meat (cod, haddock, plaice) excluding products like fish cakes, fish balls, fish spread and stew; wholegrain products (wholegrain bread and breakfast cereals); low-fat dairy products (skimmed- and semi-skimmed milk, and yoghurt). We analysed lean and fatty fish separately because they are specified in our dietary guidelines as sources of specific essential nutrients such as vitamin D and omega-3 fatty acids from fatty fish, and iodine from lean fish [21]. Each food group was divided into four consumption categories, which were roughly based on serving sizes, dietary advice, or multiples thereof. Cut-off points for each food group are given in the tables where the categorical analyses are presented (Table 2).

### Assessment of covariates

The following covariates were included in the analysis: physical activity, body mass index (BMI), smoking status, education, and intake of energy, alcohol and processed red meat.

Physical activity level was included based on validated self-report on a ten-point scale estimating physical activity at home, at work, exercising and walking, and was categorised as low (1–4 points), medium (5–6 points) or high (7–10 points) [22].

BMI (kg/m^2^) was calculated based on self-reported height and weight and has been found to provide valid ranking of BMI in NOWAC [23]. BMI was categorised in four categories: < 20, 20–24.9, 25–29.9, ≥ 30 kg/m^2^.

The smoking variable was computed by combining information on smoking status (never, former, and current), with age at smoking initiation for those who have ever smoked and additionally information of pack years for current smokers who started smoking < 20 years of age. Smoking exposure was then divided into six categories: never smoker, current heavy smoker (smoking 20 or more cigarettes per day since smoking initiation) early starter (age at start smoking < 20), current moderate smoker (smoking less than 20 cigarettes per day since smoking initiation) early starter, current smoker late starter (age at start smoking ≥ 20), former smoker early starter, former smoker late starter.

Education level was based on self-reported years of schooling and was divided into three categories (< 10, 10–12, > 12 years of schooling). Energy intake (kJ per day) was included in the analyses as a continuous variable excluding energy from alcohol. Intake of alcohol was included as a categorical variable as a group of non-consumers and two categories representing low and higher intake (g/day): non-consumers, 0–5, > 5. Intake of processed red meat included meatballs, hamburgers, sausages, and sandwich meats (e.g., liver pâté), and was divided into four categories (g/day): < 15, 15–29, 30–44, ≥ 45.

As a common procedure for dietary analyses in the NOWAC study, subcohorts (*n* = 5) were included in the analyses [18]. Subcohorts were constructed by grouping together the FFQs that were most similar as some dietary questions have been added to the FFQ due to new products available on the market, improvements of the questionnaire and specific hypotheses, and which were completed closest together in time, as the data were collected over a period of almost ten years.

### Outcome

The women were followed from return of the FFQ and until death or censoring, which was the date of emigration or end of follow-up on 31 December 2018. The source for death record linkage was the Norwegian Cause of Death Registry, which is the official cause of death statistics for Norway issued by the Norwegian Institute of Public Health [24].

### Statistical methods

We present the distribution of covariates for the lowest and the highest consumption categories of the Nordic food groups, as mean (and standard deviation) for age, as median intake (and 10th–90th percentile) for energy, and percentages (%) for the covariates expressed categorically. Spearman’s rank-order correlation was used to test the associations between the intake of the Nordic food groups. Cox proportional hazards regression models, with age as the underlying time scale, were used to examine the associations between consumption of the five Nordic food groups and all-cause mortality. The proportional hazards assumption was tested with a Schoenfeld residuals test.

Covariates included in the analysis were chosen based on the literature and selected with the use of Directed Acyclic Graphs (DAGs) (Supplemental Fig. 1) [25]. Factors known to be associated with mortality such as smoking, physical activity, BMI, intake of alcohol, intake of processed red meat and education, were included risk factors in the DAG. In addition, total energy intake and central comorbidities were included in the DAG. We constructed two different models, one adjusted for age and one multivariable-adjusted model.

**Fig. 1:**
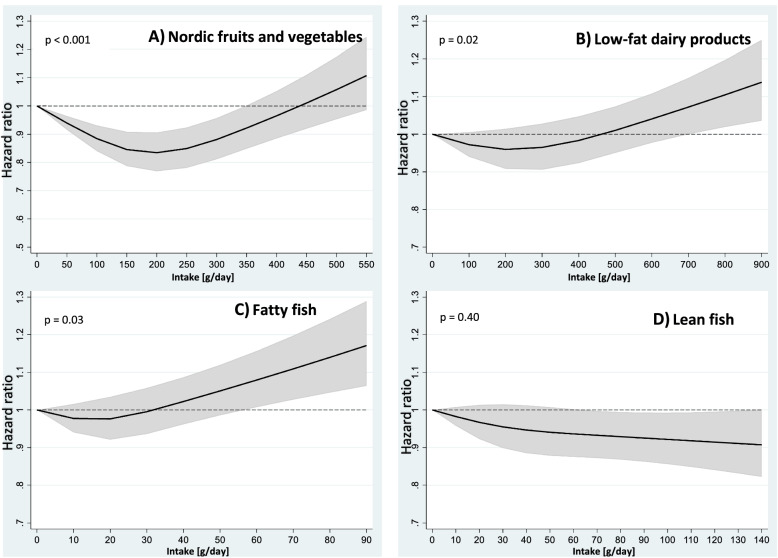
Intake of Nordic food groups and all-cause mortality by restricted cubic spline regression. From: Non-linear associations between healthy Nordic foods and all-cause mortality in the NOWAC study: a prospective study. Nordic food groups modeled by restricted cubic splines with 3 knots at percentiles 10%, 50% and 90% (Nordic fruits and vegetables 57; 164; 336. Low-fat dairy products 0; 138; 550. Fatty fish 0; 13; 35. Lean fish 0; 24; 66 g/day). Black line hazard ratio, grey area 95% confidence interval. Mutually adjusted for the healthy Nordic food groups, age (underlying timescale), BMI < 20, 20–24.9, 25–29.9, ≥ 30 (kg/m2), physical activity (low, medium, high), smoking status (never, current heavy smoker early starter, current moderate smoker early starter, current smoker late starter, former smoker early starter, former smoker late starter), education (< 10, 10–12, > 12 years of schooling), intake of energy (kJ/day continuous), alcohol (non-consumer, 0–5, > 5 g/day), and processed red meat (< 15, 15–29, 30–44, ≥ 45 g/day), stratified by subcohorts (n = 5)

The multivariable model was adjusted for age, the healthy Nordic food groups (mutually adjusted), physical activity, BMI group, smoking status, education, intake of energy, alcohol, and processed red meat. Both models examined the Nordic food groups expressed as categorical exposures, and four of the Nordic food groups were further examined in the multivariable-adjusted model as continuous exposures with restricted cubic splines. The wholegrain products variable could not be examined with restricted cubic splines because it is only based on two FFQ frequency questions and the distribution of values could not be approximated to a continuous variable.

The number of knots in the restricted cubic splines was determined by testing and comparing models with three, four and five knots according to the Akaike and Bayesian information criteria to compare how well the different models fit the data. Models with the smallest AIC value were judged to fit the data better, resulting in three knots at fixed percentiles (10, 50, 90) of the distribution [26]. The p-value for non-linearity in the restricted cubic spline analysis was calculated by performing a Wald test of the null hypothesis that the coefficient of the second spline was equal to zero. In all models, subcohorts (*n* = 5) were included as a stratum variable.

Previous analyses in NOWAC have shown associations between dietary patterns and smoking habits [27]. We therefore explored potential interactions between the Nordic food groups and smoking habits, by adding product terms in the mutually adjusted categorical models and performing likelihood-ratio tests to compare model fit between the models with and without these terms. If a statistically significant interaction effect was observed, we performed separate analyses for never and ever smokers.

We performed various sensitivity analyses. To minimise the chance of reverse causation (by including women who were ill and therefore had changed their food habits) we started follow-up two years after enrolment. As findings for Nordic fruits and vegetables in part could reflect the influence of the consumption of other fruits and vegetables [28], we made further adjustments including other fruits and vegetables in the multivariable-adjusted model. We decided to include BMI as a confounding factor even though BMI may be considered a mediating factor between diet and health outcomes. The reason for this was that the relationship between BMI and reported food intake measured at one time point is difficult to determine, and over- and under-reporting of different food groups has been related to BMI status [29]. As a sensitivity analysis, we tested omitting BMI in the multivariable-adjusted model for the categorical analyses (Supplemental Table 3). A *p*-value < 0.05 was considered statistically significant. The statistical analyses were performed using Stata / MP 16.0.

## Results

### Descriptive

During a median of 20.0 (range 0.0–22.6) years of follow-up, 8 507 women died, mainly from cancer (ICD-10 codes C00-C97) (*n* = 4 469) and cardiovascular diseases (ICD-10 codes I00-I99) (*n* = 1 538). Table 1 shows the number of participants, number of deaths, median intake of the Nordic foods, and the distribution of the covariates in the highest and lowest categories of the Nordic foods Table 1.

**Table 1:** Population distribution, intake of Nordic food groups and baseline information according to intake categories of Nordic food groups. From: Non-linear associations between healthy Nordic foods and all-cause mortality in the NOWAC study: a prospective study

Nordic food groups	Nordic fruits and vegetables		Wholegrain products		Fatty fish		Lean fish		Low-fat dairy products	
Lowest and highest intake categories (g/day)	** < 100**	** ≥ 300**	** < 60**	** ≥ 180**	** < 5**	** ≥ 30**	** < 15**	** ≥ 45**	**Non-consumers**	** ≥ 400**
Median intake (g/day)	65	368	34	180	0	42	6	61	0	550
Number of women	20 537	11 727	14 724	28 435	23 792	11 921	28 254	18 012	13 916	16 702
Number of deaths	2 530	1 022	1 419	2 869	2 497	1 403	2 529	2 486	1 554	1 992
**Covariates**										
**Age, mean (SD)**	51.2 (6.6)	52.2 (5.9)	52.1 (6.1)	51.1 (6.5)	51.0 (6.5)	53.1 (6.4)	51.0 (6.1)	53.4 (6.9)	51.7 (6.3)	51.5 (6.9)
**Physical activity n (%)**										
Low	6 811 (33)	2,500 (21)	4 494 (30)	6 918 (24)	6 984 (29)	2 872 (24)	8 076 (29)	4 672 (26)	4 202 (30)	4 374 (26)
Medium	8 582 (42)	4 752 (41)	6 027 (41)	12 594 (44)	10 138 (43)	4 919 (41)	11 744 (41)	7 690 (43)	5 703 (41)	7 256 (44)
High	5 144 (25)	4 475 (38)	4 203 (29)	8 923 (32)	6 670 (28)	4 130 (35)	8 434 (30)	5 650 (31)	4 011 (29)	5 072 (30)
**BMI n (%)**										
< 20	1 686 (8)	606 (6)	900 (6)	2 348 (8)	1 690 (7)	705 (6)	2 070 (7)	962 (5)	1 212 (9)	981 (6)
20–24.9	11 336 (55)	5 948 (51)	7 442 (51)	16 232 (57)	12 690 (53)	6 285 (53)	15 478 (55)	9 181 (51)	7 472 (54)	9 179 (55)
25–29.9	5 666 (28)	3 869 (33)	4 771 (32)	7 513 (27)	7 164 (30)	3 701 (31)	8 143 (29)	5 924 (33)	3 912 (28)	5 026 (30)
≥ 30	1 849 (9)	1 304 (11)	1 611 (11)	2 342 (8)	2 248 (10)	1 230 (10)	2 563 (9)	1 945 (11)	1 320 (9)	1 516 (9)
**Smoking status n (%)**										
Never	6 452 (31)	4 477 (38)	4 434 (30)	10 820 (38)	8 263 (35)	4 078 (34)	9 846 (35)	6 078 (34)	4 434 (30)	10 820 (38)
Current heavy smoker early starter	2 129 (10)	508 (5)	1 453 (10)	1 719 (6)	1 686 (7)	885 (7)	2 209 (8)	1 227 (7)	1 453 (10)	1 719 (6)
Current moderate smoker early starter	3 365 (16)	1 090 (9)	2 165 (15)	3 784 (13)	3 515 (15)	1 431 (12)	3 770 (13)	2 366 (13)	2 165 (15)	3 784 (13)
Current smoker late starter	2 408 (12)	875 (8)	1 338 (9)	2 753 (10)	2 371 (10)	1 296 (11)	2 373 (9)	2 280 (13)	1 338 (9)	2 753 (10)
Former smoker early starter	3 961 (19)	3 217 (27)	3 821 (26)	5 885 (21)	5 122 (21)	2 677 (23)	6 902 (24)	3 439 (19)	3 821 (26)	5 885 (21)
Former smoker late starter	2 222 (11)	1 560 (13)	1 513 (10)	3 474 (12)	2 835 (12)	1 554 (13)	3 154 (11)	2 622 (14)	1 513 (10)	3 474 (12)
**Education n (%)**										
< 10 years	5 882 (29)	2 284 (19)	3 530 (24)	7 062 (25)	6 930 (29)	2 848 (24)	5 562 (20)	6 655 (37)	3 801 (27)	4 440 (26)
10–12 years	7 258 (35)	3 953 (34)	5 345 (36)	9 432 (33)	8 664 (36)	3 797 (32)	9 911 (35)	6 057 (34)	5 017 (36)	5 813 (35)
> 12 years	7 397 (36)	5 490 (47)	5 849 (40)	11 941 (42)	8 198 (35)	5 276 (44)	12 781 (45)	5 300 (29)	5 098 (37)	6 449 (39)
**Alcohol n (%)**										
Non-consumers	4 447 (22)	2 415 (21)	2 646 (18)	6 914 (24)	6 403 (27)	2 030 (17)	5 241 (19)	4 556 (25)	3 218 (23)	3 949 (24)
0–5 (g/d)	11 029 (54)	6 590 (56)	7 981 (54)	15 621 (55)	12 991 (55)	6 203 (52)	15 235 (54)	9 971 (56)	7 156 (51)	9 159 (55)
> 5 (g/d)	5 061 (24)	2 722 (23)	4 097 (28)	5 900 (21)	4 398 (18)	3 688 (31)	7 778 (27)	3 485 (19)	3 542 (26)	3 594 (21)
**Energy P50 (P10–P90) MJ/d**	6.1 (4.1–8.5)	7.5 (5.4–10.3)	5.5 (3.6–8.1)	7.9 (6.1–10.3)	6.4 (4.3–8.9)	7.5 (5.3–10.2)	6.4 (4.3–8.8)	7.3 (5.1–10.0)	6.2 (4.1–8.8)	7.6 (5.6–10.1)
**Processed red meat intake n (%)**										
< 15 (g/d)	4 034 (20)	2 903 (25)	3 599 (24)	4 269 (15)	4 538 (19)	2 852 (24)	6 328 (23)	3 540 (20)	2 948 (21)	2 599 (16)
15–29 (g/d)	6 135 (30)	3 311 (28)	4 493 (31)	7 379 (26)	6 693 (28)	3 491 (29)	8 236 (29)	5 293 (29)	3 905 (28)	4 718 (28)
30–44 (g/d)	5 029 (24)	2 676 (23)	3 502 (24)	7 274 (26)	5 986 (25)	2 743 (23)	6 827 (24)	4 369 (24)	3 287 (24)	4 472 (27)
≥ 45 (g/d)	5 339 (26)	2 837 (24)	3 130 (21)	9 513 (33)	6 575 (28)	2 835 (24)	6 863 (24)	4 810 (27)	3 776 (27)	4 913 (29)

Percentage distribution by columns

*SD* standard deviation

*g/day* gram per day

*MJ/d* mega Joule per day

*P50* median intake, *P10* the 10 ^th^ percentile, *P90* the 90 ^th^ percentile

The oldest women were in the high-consumption group of lean and fatty fish. Within the other Nordic food groups, the age differences between categories were minimal. We found a general tendency of women in the high-consuming categories within the Nordic food groups being more physically active, and more likely to be never smokers except among high consumers of lean and fatty fish. Across all food groups, energy intake was higher in the high-consumption categories. The proportions of women reporting overweight (BMI 25.0–29.9 kg/m^2^) and obesity (BMI ≥ 30 kg/m^2^) were higher among high consumers of Nordic fruits and vegetables, whereas the opposite was observed within the wholegrain products group. Women in the highest consumption groups generally had higher education, except from the food group lean fish, where we see a higher proportion of women with low education in the highest intake category.

The highest correlation coefficient between the intake of the different Nordic food groups was found between lean and fatty fish, but the correlation was still quite low *r*_*s*_ = 0.21 (Supplemental Table 1).

### Categorical analyses for all Nordic food groups

Table 2 describes all-cause mortality according to intake categories of the Nordic food groups. Consumption of Nordic fruits and vegetables in all intake categories higher than < 100 g/day was associated with lower mortality in the age-adjusted model, but when further adjusted in the multivariable-adjusted model, it was only intake of 100–199 g/day compared to < 100 g/day that remained significant (HR 0.91 (95% CI: 0.87–0.96)). For fatty fish, the intake of 15–29 g/day compared to < 5 g/day was associated with reduced mortality in the age-adjusted model, but after further adjustments in the multivariable-adjusted model, consumption of fatty fish was no longer associated with mortality. Intake of lean fish ≥ 45 g/day compared to < 15 g/day reduced all-cause mortality (HR 0.93 (95% CI: 0.88–0.99)), and a linear trend over categories was found (*P* = 0.04). For low-fat dairy products, an intake of < 200 g/day compared to non-consumption was associated with reduced mortality in the multivariable-adjusted model (HR 0.91 (95% CI: 0.85–0.96). Increased intake of wholegrain products was associated with lower mortality in the multivariable-adjusted model (*P* for trend over categories = 0.02).

**Table 2:** Hazard ratios (HR) and all-cause mortality according to intake categories of healthy Nordic food groups. From: Non-linear associations between healthy Nordic foods and all-cause mortality in the NOWAC study: a prospective study

Healthy Nordic food groups	Intake categories (g/day)	Total N	No. of deaths	All-cause mortality		
				Age-adjusted*	Multivariable-adjusted model**	**P for trend**
				HR (95% CI)	HR (95% CI)	
**Nordic fruits and vegetables**	< 100	20 537	2 530	1.00	1.00	0.94
	100–199	32 501	3 168	0.79 (0.75–0.83)	0.91 (0.87–0.96)	
	200–299	18 904	1 787	0.77 (0.72–0.82)	0.96 (0.90–1.02)	
	≥ 300	11 727	1 022	0.78 (0.73–0.84)	1.00 (0.91–1.08)	
**Wholegrain products**	< 60	14 724	1 419	1.00	1.00	0.02
	60–119	24 439	2 669	0.91 (0.85–0.97)	0.96 (0.90–1.03)	
	120–179	16 071	1 550	0.78 (0.73–0.83)	0.91 (0.84–0.98)	
	≥ 180	28 435	2 869	0.84 (0.79–0.90)	0.89 (0.82–0.97)	
**Fatty fish**	< 5	23 792	2 497	1.00	1.00	0.17
	5–14	25 882	2 517	0.94 (0.89–1.00)	1.01 (0.95–1.07)	
	15–29	22 074	2 090	0.90 (0.85–0.96)	0.99 (0.93–1.05)	
	≥ 30	11 921	1 403	0.98 (0.92–1.05)	1.06 (0.99–1.14)	
**Lean fish**	< 15	28 254	2 529	1.00	1.00	0.04
	15–29	22 562	2 023	0.92 (0.87–0.97)	0.96 (0.91–1.02)	
	30–44	14 841	1 469	0.93 (0.87–0.99)	0.99 (0.92–1.05)	
	≥ 45	18 012	2 486	0.95 (0.90–1.01)	0.93 (0.88–0.99)	
**Low-fat dairy products**	Non-consumers	13 916	1 554	1.00	1.00	0.14
	< 200	34 848	3 078	0.79 (0.74–0.84)	0.91 (0.85–0.96)	
	200–399	18 203	1 883	0.78 (0.73–0.84)	0.96 (0.90–1.03)	
	≥ 400	16 702	1 992	0.84 (0.78–0.90)	0.99 (0.92–1.06)	

*HR* hazard ratio, *CI* confidence interval

^*^Age-adjusted with age as underlying timescale and subcohorts (n = 5) included as strata variable

^**^Age-adjusted and mutually adjusted for the healthy Nordic food groups, BMI < 20, 20–24.9, 25–29.9, ≥ 30 (kg/m^2^), physical activity (low, medium, high), smoking status (never, current heavy smoker early starter, current moderate smoker early starter, current smoker late starter, former smoker early starter, former smoker late starter), education (< 10, 10–12, > 12 years of schooling) intake of energy (kJ/day continuous), alcohol (non-consumer, 0–5, > 5 g/day), and processed red meat (< 15, 15–29, 30–44, ≥ 45 g/day)

### Restricted cubic spline regression analyses

The restricted cubic spline regression analyses showed a significant J-shaped association for the food groups Nordic fruits and vegetables (Fig. 1A), low-fat dairy products (Fig. 1B) and fatty fish (Fig. 1C), but not for lean fish (Fig. 1D) Fig. 1 (Additional file 1).

For Nordic fruits and vegetables, the nadir (the intake level associated with lowest mortality) was observed at 200 g/day (HR 0.83 (95% CI: 0.77–0.91) compared to no consumption) (Fig. 1A). For low-fat dairy products, the nadir was observed at 200 g/day (HR 0.96 (95% CI: 0.91–1.01) compared to no consumption. Consumption of low-fat dairy products ≥ 800 g/day compared to no consumption increased mortality (Fig. 1B). For fatty fish, the nadir was observed at an intake level of 10–20 g/day (20 g/day: HR 0.98 (95% CI: 0.92–1.03)), but the mortality was not significantly lower than for not consuming fatty fish at all (Fig. 1C). Excessive consumption, on the other hand, was associated with increased mortality from 60 g/day (HR 1.08 (95% CI: 1.01–1.16)). For lean fish, we observed that increased intake reduced mortality, and that intake between 80–110 g/day was statistically significantly associated with all-cause mortality (80 g/day: HR 0.93 (95% CI: 0.87–0.99)) (Fig. 1D).

### Intake of Nordic fruits and vegetables and mortality in never and ever smokers

We observed a significant interaction between smoking status and Nordic fruits and vegetables regarding all-cause mortality, and thus separate analyses for never and ever smokers are also presented. The median consumption of Nordic fruits and vegetables was 173 g/day (P10: 65 g/day, P90: 342 g/day) in never smokers, and 159 g/day (P10: 53 g/day, P90: 332 g/day) in ever smokers (Supplemental Table 2).

**Fig. 2:**
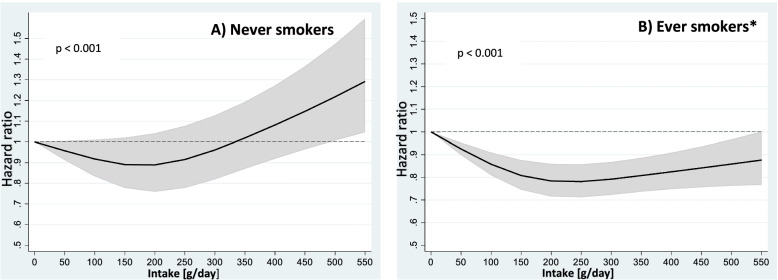
Intake of Nordic fruits and vegetables and all-cause mortality by restricted cubic splines stratified by never and ever smokers. From: Non-linear associations between healthy Nordic foods and all-cause mortality in the NOWAC study: a prospective study. Nordic fruits and vegetables modeled by restricted cubic splines with 3 knots at percentiles 10%, 50% and 90% (Never smokers: 65; 173; 343. Ever smokers: 53; 160; 332). Black line hazard ratio, grey area 95% confidence interval. Age-adjusted and mutually adjusted for the healthy Nordic food groups, BMI < 20, 20–24.9, 25–29.9, ≥ 30 (kg/m2), physical activity (low, medium, high), education (< 10, 10–12, > 12 years of schooling), intake of energy (kJ/day continuous), alcohol (non-consumer, 0–5, > 5 g/day), and processed red meat (< 15, 15–29, 30–44, ≥ 45 g/day). *Additionally, adjusted for pack-years

In the categorical analysis, intake between 100–199 g/day compared to < 100 g/day was associated with reduced mortality among never smokers with similar strength as in the unstratified analysis (HR 0.89 (95% CI 0.81–0.99). However, for ever smokers, increased intake was associated with lower mortality in the multivariable-adjusted model (*P* for trend over categories < 0.001) (Table 3). In the restricted cubic spline regressions, the observed association was only significant in ever smokers with the nadir at 200–250 g/day (HR 0.79 (95% CI: 0.72–0.87). In never smokers, the nadir was observed at 150–200 g/day (150 g/day: HR 0.89 (95% CI: 0.78–1.02); 200 g/day: HR 0.89 (95% CI: 0.76–1.05) (Fig. 2). Furthermore, consumption of Nordic fruits and vegetables > 500 g/day increased mortality among never smokers, but there were only 33 deaths registered at this consumption level Fig. 2 (Additional file 2).

**Table 3:** Hazard ratios (HR) and all-cause mortality according to intake categories of Nordic fruits and vegetables stratified by smoking status. From: Non-linear associations between healthy Nordic foods and all-cause mortality in the NOWAC study: a prospective study

**All-cause mortality**								
	**Never smokers**				Ever smokers*			
**Intake categories of Nordic fruits and vegetables (g/day)**	**Total N**	**No. of deaths**	**HR (95% CI)**	**P for trend**	**Total N**	**No. of deaths**	**HR (95% CI)**	**P for trend**
** < 100**	6 452	588	1.00	0.10	14 085	1 942	1.00	< 0.001
**100–199**	11 654	905	0.89 (0.81–0.99)		20 847	2 263	0.86 (0.80–0.91)	
**200–299**	7 232	605	1.03 (0.91–1.15)		11 672	1 182	0.82 (0.76–0.89)	
** ≥ 300**	4 477	333	1.07 (0.93–1.24)		7 250	689	0.84 (0.76–0.92)	

*HR* hazard ratio, *CI* confidence interval

Age-adjusted and mutually adjusted for the healthy Nordic food groups, BMI < 20, 20–24.9, 25–29.9, ≥ 30 (kg/m^2^), physical activity (low, medium, high), education (< 10, 10–12, > 12 years of schooling), intake of energy (kJ/day continuous), alcohol (non-consumer, 0–5, > 5 g/day), and processed red meat (< 15, 15–29, 30–44, ≥ 45 g/day)

^*^Additionally, adjusted for pack-years

### Sensitivity analyses

Sensitivity analysis, starting follow-up two years after enrolment excluding 350 cases, did not change the results (Supplementary Fig. 2). Further adjustments including other fruits and vegetables in the multivariable-adjusted model did not influence the results (Supplementary Fig. 3). Omitting BMI in the multivariable-adjusted categorical model did not lead to changes in the results (Supplemental Table 3).

## Discussion

We observed a J-shaped trend between intake of Nordic fruits and vegetables, fatty fish and low-fat dairy products and all-cause mortality, implying that with increasing intake of some traditional Nordic food groups, mortality might change in a non-linear fashion. As the null hypothesis of linearity was not rejected for lean fish, we conclude that the non-linear components did not add more information to those data than a linear model. For wholegrain products, our results were limited to categorical analysis, but a test for trend over categories pointed to a linear association with mortality.

The restricted cubic splines allow for predictions for any value of the variable, compared to only four probabilities in our categorical analyses, or compared to the alternative of modelling a linear relationship. Thus, the estimates from the splines add more information to the results and are therefore emphasized. The results from both modelling tools point in the same direction, but the effect estimates associated with the nadir from the restricted cubic spline models show a stronger negative association for Nordic fruits and vegetables, and a weaker negative association for low-fat dairy products than what we observed in the categorical analyses. However, as most self-reported dietary assessment methods are better suited for ranking than estimating absolute intake, the absolute consumption levels found to be associated with the lowest mortality in this study, as shown in Table 2 and the figures, are probably not as important as the shape of the curves.

The maximum benefit of consuming Nordic fruits and vegetables was achieved at around 200 g/day, which is below the recommended intake of all fruits and vegetables of five servings per day [21]. Optimal health benefits of fruit and vegetable consumption achieved at a more modest intake level than currently recommended (around three to four servings per day) have also been found in the PURE study [30]. Non-linear inverse associations of fruit and vegetable intake with all-cause mortality have been shown in previous meta-analyses [31, 32], but with dose–response curves that differed from our J-shaped curve for Nordic fruits and vegetables. Aune et al. found that the benefit of increasing fruits and vegetables intake was larger at lower intake levels but observed reductions of risk up to 800 g/day [32], while Wang et al. found that the benefit of fruits and vegetables plateaued at approximately 5–6 servings per day [31].

The benefit of consuming Nordic fruits and vegetable seemed stronger in ever- than in never-smokers. Similar tendencies were reported in the European Prospective Investigation into Cancer and Nutrition, which also included a subsample of women from NOWAC [33]. In addition, a meta-analysis of prospective cohort studies on the association between consumption of fruits and vegetable and risk of lung cancer found stronger associations with lung cancer among smokers. Antioxidant properties of fruits and vegetables are protective against increased oxidative stress caused by smoking [34].

The impact of dairy intake on mortality has been extensively studied, but results are not conclusive [35, 36]. The divergence of results could be due to variation between the different types of dairy products being investigated (i.e., total dairy, specific categories of dairy such as milk, yoghurt, cheese, low-fat/high-fat dairy), different cut-off points between studies, but also the quality of the underlying diet in different populations. Still, when comparing results on low-fat milk consumption as a specific dairy category and mortality in Nordic populations, one study finds an increased mortality [37] while another found no association [38]. It is noted that the fat content in yoghurt, which was part of the low-fat dairy products in the present study, could be up to 3.4%, and therefore not necessarily considered low-fat. Hence, our results are not directly comparable with these studies. Our analysis showed a non-linear association with low-fat dairy and mortality, much in line with what Ding et al. found for total dairy consumption in three prospective cohort studies in women and men [39].

We observed that consumption up to the recommended 200 g of fatty fish/week (29 g/day) was within a non-significant beneficial range, but when intake reached 60 g/day there was a significantly increased mortality. In contrast, higher consumption of lean fish reduced all-cause mortality. Several large cohort studies have not been able to show any reduced mortality linked to frequent fish consumption [40, 41], but some protective associations are found in metaanlyses [42–44]. Engeset et al. found a non-linear trend with fatty fish consumption and mortality in the European Prospective investigation into Cancer and Nutrition cohort, which included a part of our sample [41]. Also, a study on fish consumption and mortality in a cohort of Swedish men and women found a U-shaped association between consumption of fish and all-cause mortality, which was more pronounced in women [45]. Furthermore, when they considered lean and fatty fish separately, they found no associations between consumption of lean fish and mortality, but up to 68% increased mortality in women who consumed 50 g/day fatty fish compared to the median intake level (9 g/day).

Even though fish is a good source of essential nutrients, it is also a source of environmental contaminants such as dioxins, which are classified as carcinogens, and accumulates in the adipose tissue [21, 46, 47]. While lean fish store fat in the liver, fatty fish store it in the fillet itself, which then contains more of these substances compared to lean fish. One can speculate whether this is related to the observed increased mortality associated with high consumption of fatty fish, but not with lean fish.

The observed protective effect of wholegrain products on all-cause mortality in the present analysis is supported by meta-analyses of prospective cohort studies including populations from the US, Europe, and Asia [48, 49]. In the meta-analysis by Aune et al., reductions in mortality for whole grains were observed up to an intake of 225 g per day and they found a steeper reduction at lower intake levels. In a study on Norwegian wholegrain eaters by Jacobs et al. included in the meta-analyses, they found an inverse association between a calculated wholegrain consumption score and mortality, with the highest score being most beneficial [50]. This score was calculated based on slices of bread multiplied by percentages of wholegrain and was thus based on more detailed information on wholegrain consumption than was available in the present study.

These findings imply that if linear associations between traditional Nordic foods and health outcomes are assumed, it might lead to wrong conclusions as the relationships can be non-linear. Furthermore, they imply that lean and fatty fish might be differently associated to health outcomes, and that this aspect therefore should be investigated further in future studies. Also, the search for optimal intake levels of traditional foods should be emphasised in further studies on regional sustainable diets, both for health and to reduce the burden of food production on the environment.

Establishing optimal intake levels of foods for health is, however, not straightforward, given the limitations inherent in FFQs to provide precise estimates of actual food intake. Furthermore, analyses on isolated foods does not consider synergistic and antagonistic interactions between food groups existing within the same diet, and possibly also with other lifestyle factors, which might explain why isolated foods sometimes show a seemingly confusing pattern on health. These interactions might be better captured with dietary pattern analyses, but as indicated by our results, careful consideration on how to score individual foods in construction of a combined diet score is warranted.

### Strengths and limitations

The strengths of this study include a large sample size, a high number of deaths and the long follow-up (median 20 years), providing enough statistical power in the analysis. Linkage to registry is a strength as all deaths are confirmed. Furthermore, the risk of sampling bias is considered low due to the selection of women through the National Registry. Another strength is that a validated questionnaire was used to assess food intake and covariates [15–17, 22, 23].

The study is, however, limited by having only one assessment of diet, as dietary habits probably have changed during follow-up. Recalling the habitual diet with the use of FFQ over the past year could be challenging and give rise to misclassification of dietary exposures, but this is expected to be non-differential. In addition, the FFQ was not designed to measure all foods that are part of a healthy Nordic diet and hence does not capture all relevant food components such as wild berries and vegetables like kale or distinguish between specific varieties of Nordic wholegrains such as rye and barley. Furthermore, precise assessment of dietary exposure is difficult and measurement errors are inevitable in nutritional epidemiology. Also, even though we adjusted for covariates that were unevenly distributed across intake categories of the Nordic food groups, residual confounding due to imprecise assessment of these factors as well as unmeasured factors is likely. The results must be interpreted with caution as the moderate consumers are probably more representative of what most people eat, while both low and high consumers can be different in many ways (e.g., extreme dieters, vegans, people with allergies).

## Conclusion

Nordic fruits and vegetables, low-fat dairy products and fatty fish was non-linearly associated to all-cause mortality, while increased intake of lean fish and wholegrain products reduced all-cause mortality among middle-aged and older women.

While high consumption of fatty fish increased all-cause mortality, the opposite was found for lean fish, suggesting that they should not be treated as one food group in relation to health outcomes.

Consumption of Nordic fruits and vegetables was most beneficial in women that were either current or former smokers, implying that dietary interventions might be especially important for women with higher risk of premature death due to smoking. Our results indicate that more attention to nonlinear associations is warranted in analyses of diet and health-outcomes.

## Supplementary Information

**Additional file 1.**

**Additional file 2.**

**Additional file 3: Table 1.** Spearman correlation coefficients between intake of Nordic food groups. From: Non-linear associations between healthy Nordic foods and all-cause mortality in the NOWAC study: a prospective study. **Table 2.** Population distribution and intake of Nordic fruits and vegetables stratified by never and ever smokers. From: Non-linear associations between healthy Nordic foods and all-cause mortality in the NOWAC study: a prospective study. **Table 3.** Hazard ratios (HR) and all-cause mortality according to intake categories of Nordic food groups leaving BMI out of the multivariable-adjusted model. From: Non-linear associations between healthy Nordic foods and all-cause mortality in the NOWAC study: a prospective study. **Figure 1.** DAG constructed for the analyses for estimating the total effect of Nordic foods on all-cause mortality. From: Non-linear associations between healthy Nordic foods and all-cause mortality in the NOWAC study: a prospective study. **Figure 2.** Intake of Nordic food groups and all-cause mortality by restricted cubic spline regression excluding death cases that occurred in first two years of follow-up. From: Non-linear associations between healthy Nordic foods and all-cause mortality in the NOWAC study: a prospective study. **Figure 3.** Intake of Nordic fruits and vegetables and all-cause mortality by restricted cubic spline regression, estimates further adjusted for other fruits and vegetables. From: Non-linear associations between healthy Nordic foods and all-cause mortality in the NOWAC study: a prospective study.

## Data Availability

The datasets generated and/or analysed during the current study are not publicly available due to restrictions that apply to the availability of these data, which were used under licence for the current study, but are available from the corresponding author on reasonable request.

## Abbreviations

NOWAC: The Norwegian Women and Cancer Study
BMI: Body mass index
FFQ: Food frequency questionnaire
DAGs: Directed Acyclic Graphs
HR: Hazard ratio
CI: Confidence interval
PURE study: The Prospective Urban Rural Epidemiology study
REK: The Regional Committee for Medical and Health Research Ethics
