# Supplementary material for: Non-linear associations between healthy Nordic foods and all-cause mortality in the NOWAC study: a prospective study
Source: BMC Public Health. 2022 Jan 25;22:169. doi: 10.1186/s12889-022-12572-8 (PMC8788118; doi:10.1186/s12889-022-12572-8)
Supplement: Supplementary file 1 — Additional file 1. [file 12889_2022_12572_MOESM1_ESM.docx]

|  |   **Intake [g/day]**  Hazard ratio  p < 0.001  **A) Nordic fruits and vegetables** |   Hazard ratio  **Intake [g/day]**  p = 0.02  **B) Low-fat dairy products** |
| --- | --- | --- |
|  |   **C) Fatty fish**  Hazard ratio  p = 0.03 |   **Intake [g/day]**  Hazard ratio  p = 0.40  **D) Lean fish** |
|  | **Intake [g/day]** | |
