# Supplementary material for: Non-linear associations between healthy Nordic foods and all-cause mortality in the NOWAC study: a prospective study
Source: BMC Public Health. 2022 Jan 25;22:169. doi: 10.1186/s12889-022-12572-8 (PMC8788118; doi:10.1186/s12889-022-12572-8)
Supplement: Supplementary file 2 — Additional file 2. [file 12889_2022_12572_MOESM2_ESM.docx]

|   p < 0.001  **A) Never smokers**  **Intake [g/day**] |   p < 0.001  **B) Ever smokers***  Hazard ratio  **Intake [g/day]** |
| --- | --- |

Hazard ratio
