# Supplementary material for: Non-linear associations between healthy Nordic foods and all-cause mortality in the NOWAC study: a prospective study
Source: BMC Public Health. 2022 Jan 25;22:169. doi: 10.1186/s12889-022-12572-8 (PMC8788118; doi:10.1186/s12889-022-12572-8)
Supplement: Supplementary file 3 — Additional file 3: Table 1. Spearman correlation coefficients between intake of Nordic food groups. From: Non-linear associations between healthy Nordic foods and all-cause mortality in the NOWAC study: a prospective study. Table 2. Population distribution and intake of Nordic fruits and vegetables stratified by never and ever smokers. From: Non-linear associations between healthy Nordic foods and all-cause mortality in the NOWAC study: a prospective study. Table 3. Hazard ratios (HR) and all-cause mortality according to intake categories of Nordic food groups leaving BMI out of the multivariable-adjusted model. From: Non-linear associations between healthy Nordic foods and all-cause mortality in the NOWAC study: a prospective study. Figure 1. DAG constructed for the analyses for estimating the total effect of Nordic foods on all-cause mortality. From: Non-linear associations between healthy Nordic foods and all-cause mortality in the NOWAC study: a prospective study. Figure 2. Intake of Nordic food groups and all-cause mortality by restricted cubic spline regression excluding death cases that occurred in first two years of follow-up. From: Non-linear associations between healthy Nordic foods and all-cause mortality in the NOWAC study: a prospective study. Figure 3. Intake of Nordic fruits and vegetables and all-cause mortality by restricted cubic spline regression, estimates further adjusted for other fruits and vegetables. From: Non-linear associations between healthy Nordic foods and all-cause mortality in the NOWAC study: a prospective study. [file 12889_2022_12572_MOESM3_ESM.docx]

# “Non-linear associations between foods basic in a healthy Nordic diet and all-cause mortality in the Norwegian Women and Cancer study: a prospective cohort study”

Supplemental Tables 1-3

Supplemental Table 1. Spearman correlation coefficients between intake of Nordic food groups

From: Non-linear associations between healthy Nordic foods and all-cause mortality in the NOWAC study: a prospective study

| Healthy Nordic food groups | Nordic fruits and  vegetables intake | Wholegrain intake | Fatty fish intake | Lean fish intake | Low-fat dairy intake |
| --- | --- | --- | --- | --- | --- |
| Nordic fruits and vegetables intake | 1 |  |  |  |  |
| Wholegrain intake | 0.05 | 1 |  |  |  |
| Fatty fish intake | 0.19 | 0.01 | 1 |  |  |
| Lean fish intake | 0.15 | 0.09 | 0.21 | 1 |  |
| Low-fat dairy intake | 0.02 | 0.14 | 0.02 | 0.07 | 1 |

Supplemental Table 2. Population distribution and intake of Nordic fruits and vegetables stratified by never and ever smokers

From: Non-linear associations between healthy Nordic foods and all-cause mortality in the NOWAC study: a prospective study

| Smoking status | Total N | No. of deaths | Nordic fruits and vegetables intake  Median intake (P10-P90) (g/day) | Nordic fruits and vegetables intake categories (g/day) | | | |
| --- | --- | --- | --- | --- | --- | --- | --- |
|  |  |  |  | <100 | 100-199 | 200-299 | ≥300 |
| Never smokers | 29 815 | 2 431 | 173 (65-342) | 68 | 150 | 237 | 366 |
| Ever smokers | 53 854 | 6 076 | 160 (53-332) | 63 | 148 | 237 | 369 |

Supplemental Table 3. Hazard ratios (HR) and all-cause mortality according to intake categories of Nordic food groups leaving BMI out of the multivariable-adjusted model

From: Non-linear associations between healthy Nordic foods and all-cause mortality in the NOWAC study: a prospective study

| Healthy Nordic food groups | Intake categories (g/day) | Total N | No. of deaths | All-cause mortality | | |
| --- | --- | --- | --- | --- | --- | --- |
|  |  |  |  | Age-adjusted* | Multivariable-adjusted model ** | P for trend |
|  |  |  |  | HR (95% CI) | HR (95% CI) |  |
| Nordic fruits and vegetables | < 100  100–199  200–299  ≥ 300 | 20 537  32 501  18 904  11 727 | 2 530  3 168  1 787  1 022 | 1.00  0.79 (0.75–0.83)  0.77 (0.72–0.82)  0.78 (0.73–0.84) | 1.00  0.91 (0.86–0.96)  0.95 (0.89–1.01)  0.98 (0.91–1.06) | 0.94 |
| Wholegrain products | < 60  60–119  120–179  ≥ 180 | 14 724  24 439  16 071  28 435 | 1 419  2 669  1 550  2 869 | 1.00  0.91 (0.85–0.97)  0.78 (0.73–0.83)  0.84 (0.79–0.90) | 1.00  0.97 (0.90–1.03)  0.91 (0.85–0.99)  0.91 (0.85–0.98) | 0.02 |
| Fatty fish | < 5  5–14  15–29  ≥ 30 | 23 792  25 882  22 074  11 921 | 2 497  2 517  2 090  1403 | 1.00  0.94 (0.89–1.00)  0.90 (0.85–0.96)  0.98 (0.92–1.05) | 1.00  1.01 (0.95–1.07)  0.99 (0.93–1.05)  1.06 (0.98–1.13) | 0.17 |
| Lean fish | < 15  15–29  30–44  ≥ 45 | 28 254  22 562  14 841  18 012 | 2 529  2 023  1 469  2 486 | 1.00  0.92 (0.87–0.97)  0.93 (0.87–0.99)  0.95 (0.90–1.01) | 1.00  0.96 (0.91–1.02)  0.98 (0.92–1.05)  0.93 (0.88–0.99) | 0.04 |
| Low-fat dairy products | Non-consumers  <200  200–399  ≥400 | 13 916  34 848  18 203  16 702 | 1 554  3 078  1 883  1 992 | 1.00  0.79 (0.74–0.84)  0.78 (0.73–0.84)  0.84 (0.78–0.90) | 1.00  0.90 (0.84–0.95)  0.94 (0.88–1.01)  0.97 (0.91–1.04) | 0.14 |

* Age-adjusted with age as underlying timescale and subcohorts (n=5) included as strata variable

** Age-adjusted and mutually adjusted for the healthy Nordic food groups, physical activity (low, medium, high), smoking status (never, current heavy smoker early starter, current moderate smoker early starter, current smoker late starter, former smoker early starter, former smoker late starter), education (<10, 10-12, >12 years of schooling), intake of energy (kJ/day continuous), alcohol (non-consumer, 0–5, > 5 grams/day), processed meat (< 15, 15–29, 30–44, ≥ 45 grams/day)

Supplemental Figures 1-3

Supplemental Figure 1. DAG constructed for the analyses for estimating the total effect of Nordic foods on all-cause mortality

From: Non-linear associations between healthy Nordic foods and all-cause mortality in the NOWAC study: a prospective study


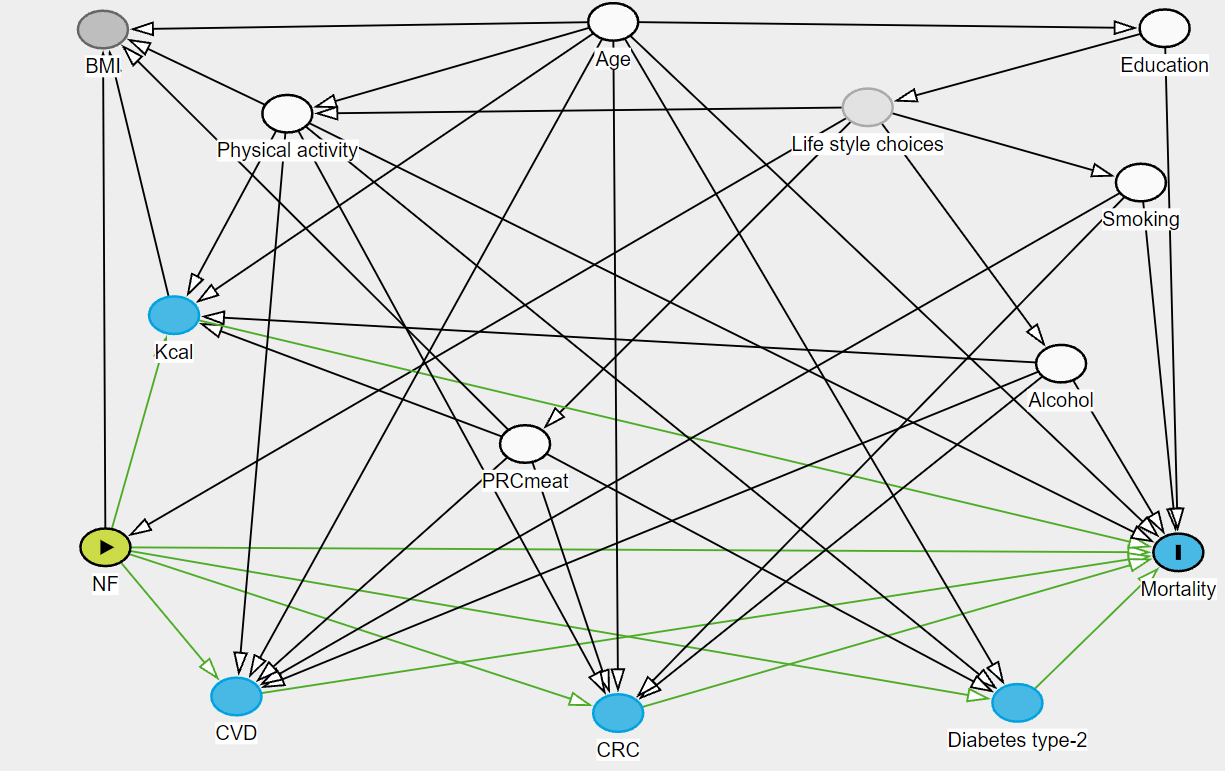


Red circle: Light grey circle: unobserved variables Blue circles: observed variables
Yellow circle: exposure
Blue circle with I: outcome
White circles: adjusted variables
NF= healthy Nordic foods CRC= colorectal cancer CVD= cardiovascular disease BMI= Body Mass Index PRCmeat= processed red meat Kcal= energy intake

The figure is created from www.dagitty.net

Supplemental Figure 2. Intake of Nordic food groups and all-cause mortality by restricted cubic spline regression excluding death cases that occurred in first two years of follow-up

From: Non-linear associations between healthy Nordic foods and all-cause mortality in the NOWAC study: a prospective study

|  |   **A) Nordic fruits and vegetables**  p < 0.001  **Intake [g/day]**  Hazard ratio |   **B) Low-fat dairy products**  **Intake [g/day]**  p = 0.07  Hazard ratio |
| --- | --- | --- |
|  |   **C) Fatty fish**  Hazard ratio  P = 0.04 |   Hazard ratio  **Intake [g/day]**  **D) Lean fish**  p= 0.47 |
|  | **Intake [g/day]** | |

Nordic food groups modeled by restricted cubic splines with 3 knots at percentiles 10%, 50% and 90% (Nordic fruits and vegetables 57;164;336. Low-fat dairy products 0;138;550. Fatty fish 0;13;35. Lean fish 0;24;66 g/day).

Black line hazard ratio, grey area 95% confidence interval

Mutually adjusted for the healthy Nordic food groups, age (underlying timescale), BMI <20, 20-24.9, 25-29.9, ≥30 (kg/m^2^), physical activity (low, medium, high), smoking status (never, current heavy smoker early starter, current moderate smoker early starter, current smoker late starter, former smoker early starter, former smoker late starter), education (<10, 10-12. >12 years of schooling), intake of energy (kJ/day continuous), alcohol (non-consumer, 0-5, >5 gram/day), and processed red meat (<15, 15-29, 30-44, ≥45 gram/day), subcohorts (n=5) included as strata variable

Supplemental Figure 3. Intake of Nordic fruits and vegetables and all-cause mortality by restricted cubic spline regression, estimates further adjusted for other fruits and vegetables

From: Non-linear associations between healthy Nordic foods and all-cause mortality in the NOWAC study: a prospective study

p < 0.001

Hazard ratio

**Intake [g/day]**

Nordic fruits and vegetables modeled by restricted cubic splines with 3 knots at percentiles 10%, 50% and 90% (57; 164; 336 g/day).

Black line hazard ratio, grey area 95% confidence interval

Mutually adjusted for the healthy Nordic food groups, age (underlying timescale), BMI < 20, 20–24.9, 25–29.9, ≥ 30 (kg/m^2^), physical activity (low, medium, high), smoking status (never, current heavy smoker early starter, current moderate smoker early starter, current smoker late starter, former smoker early starter, former smoker late starter), education (<10, 10-12, >12 years of schooling), intake of energy (kJ/day continuous), alcohol (non-consumer, 0–5, > 5 grams/day), processed red meat (< 15, 15–29, 30–44, ≥ 45 grams/day) and other fruits and vegetables (<100, 100-199, 200-299, ≥300), subcohorts (n=5) included as strata variable
